# Emerging interactions between diet, gastrointestinal helminth infection, and the gut microbiota in livestock

**DOI:** 10.1186/s12917-021-02752-w

**Published:** 2021-01-29

**Authors:** Andrew R. Williams, Laura J. Myhill, Sophie Stolzenbach, Peter Nejsum, Helena Mejer, Dennis S. Nielsen, Stig M. Thamsborg

**Affiliations:** 1 Department of Veterinary and Animal Sciences, University of Copenhagen, Copenhagen, Denmark; 2 Department of Clinical Medicine, Aarhus University, Aarhus, Denmark; 3 Department of Food Science, University of Copenhagen, Copenhagen, Denmark

**Keywords:** Livestock, Helminths, Gut microbiota, Diet, prebiotics, probiotics, immunity

## Abstract

Increasing evidence suggests that nutritional manipulation of the commensal gut microbiota (GM) may play a key role in maintaining animal health and production in an era of reduced antimicrobial usage. Gastrointestinal helminth infections impose a considerable burden on animal performance, and recent studies suggest that infection may substantially alter the composition and function of the GM. Here, we discuss the potential interactions between different bioactive dietary components (prebiotics, probiotics and phytonutrients) and helminth infection on the GM in livestock. A number of recent studies suggest that host diet can strongly influence the nature of the helminth-GM interaction. Nutritional manipulation of the GM may thus impact helminth infection, and conversely infection may also influence how the GM responds to dietary interventions. Moreover, a dynamic interaction exists between helminths, the GM, intestinal immune responses, and inflammation. Deciphering the mechanisms underlying the diet-GM-helminth axis will likely inform future helminth control strategies, as well as having implications for how health-promoting feed additives, such as probiotics, can play a role in sustainable animal production.

## Background

The world’s population is increasing at a rapid rate, and food security is crucial to meet the nutritional demands of this growing number of people. Efficient livestock production is a vital contributor to ensuring this demand is met [1]. However, the animal production sector faces intense challenges related to carbon emissions and high resource usage [2]. Efficiency is thus very important, both for environmental sustainability and for the profitability of individual farming enterprises. In this respect, ensuring healthy animals that are not encumbered by chronic pathogen infection is a key aim for promoting robust and efficient livestock systems. However, consumer concern with widespread antimicrobial drug usage and the rapid development of pathogen resistance to antibiotics and parasiticides means that existing tools to combat infection are not sustainable [3].

Dietary interventions have been proposed as a sustainable tool to ensure efficient production by suppressing pathogens, boosting immune function, and sustaining a resilient and diverse gut microbiome [4]. The composition of the prokaryotic gut microbiota (GM) has been suggested to play a key role in animal health and productivity [5]. The role of gut microorganisms in extracting nutrients from feed for host utilization has long been recognized for both ruminant and monogastric animals, but additional roles in the regulation of immunity, inflammation and general homeostasis are increasingly appreciated [6]. To this end, manipulation of the GM through prebiotic dietary supplements, or the provision of probiotic cocktails of ‘beneficial’ bacteria, has received much attention as a health-promoting strategy that may supplement or eventually replace prophylactic use of antimicrobial drugs [7].

Gastrointestinal helminths are ubiquitous in livestock, particularly those in pasture-based systems but also in indoor-based swine and poultry production [8–10]. Helminth infections are typically sub-clinical, but can suppress animal productivity and welfare through reduced weight gain and efficiency of feed utilization [11]. More recently, the effect of helminth infection on the composition of the GM has become increasingly clear. Studies in a variety of livestock species have shown that helminth infection can markedly change both the composition and predicted metabolic potential of the GM [12], suggesting that disruption of basal GM function may be a contributing factor to the decreased productivity in infected animals. Here, we briefly review key studies on helminth-GM interactions in livestock, and then discuss the emerging implications that the diet-microbiota-helminth axis may have on animal health and productivity. We consider the potential of GM-targeting dietary additives such as prebiotics as a novel anthelmintic therapy, and also whether concurrent helminth infection may modulate the beneficial effects of some putative health-promoting dietary components. Finally, we suggest some pertinent areas for future research to better understand the relationships between helminth infection and gut health in livestock.

### Gastrointestinal helminths in livestock

Helminth infection is the most serious health concern of grazing animals, particularly small ruminants [3]. Infection with the abomasal nematodes *Haemonchus contortus* or *Teladorsagia circumcincta* may cause anaemia or malnutrition, respectively, and clinical haemonchosis can be fatal for young or immuno-compromised animals [13]. Intestinal nematodes such as *Trichostrongylus* or *Cooperia* spp. can cause severe diarrhoea, or, more commonly, reduce growth rate and meat and wool production. Similarly, in cattle, the abomasal parasite *Ostertagia ostertagi* can cause chronic infection that reduce animal growth and milk production [14]. Apart from *H. contortus*, for which a vaccine is only available in few countries, control is exclusively based on treatment with a small number of chemical drug classes [3]. Resistance to all drug classes has been reported. In the older benzimidazole and macrocyclic lactone (ML) drug families resistance is extremely common, and has already been reported in the most recent class to be released (the amino-acetonitrile derivatives) [15]. Similar to the situation in ruminants, infection in grazing horses is widespread, primarily due to cyathostomins*, Parascaris equorum* and, in localised regions, *Strongylus vulgaris*. Multiple-drug resistance has been reported in cyathostomins and *P. equorum* [16].

In pigs, infections with *Ascaris suum*, *Oesophagostomum dentatum,* and *Trichuris suis* are common. This is particularly the case in outdoor production systems, which are the norm in developing countries and also comprise a small but growing proportion of pork production in developed countries [17]. *A. suum* and *O. dentatum* are also found in high numbers in indoor herds despite improved hygiene in these systems [18, 19]. *T. suis,* whilst less common in indoor herds, has the potential to cause mucohemorrhagic diarrhoea and death when intensive outbreaks occur [20]. Compared to ruminants and horses, drug resistance appears to be less common in swine helminths. However, resistance to benzimidazoles, levamisole, and the ML ivermectin has been reported in *O. dentatum,* and, given the high usage of anthelmintic drugs in conventional pig farms, resistance is likely to be either under-estimated or will likely increase in coming years [21, 22].

In addition to the direct effects of helminths on animal growth and feed efficiency, it is increasingly appreciated that infection can have indirect effects on animal health by modulation of host immunity and increased susceptibility to secondary infections. Helminths induce strongly polarized T-helper (Th)-2 type immune responses, and this polarization may suppress Th1-driven immunity which is important for combating bacterial and viral infection. For example, a concurrent bacterial infection in pigs exposed to *T. suis* may cause necrotic colitis through worm-induced suppression of immune responses to bacteria [23]. Importantly, the efficacy of vaccination against other pathogens may be compromised during helminth infection due to their strong immune-suppressive properties. This has been repeatedly demonstrated in both rodent models and also clinical studies in humans in helminth-endemic regions [24, 25], and vaccination against *Mycoplasma* in pigs is impaired by concurrent *A. suum* infection [26]. Thus, the detrimental effects of intestinal helminth infection may not be limited to the more intuitive pathological consequences on digestive function, but also more subtle influences on whole-body physiology driven by the profound manipulative properties of these parasites.

### Interactions between helminths and the gut microbiota

The effect of helminth infection on the GM in both humans and rodents has been the subject of a plethora of studies in recent years However, generalisations about the interaction between helminths and the prokaryotic GM are problematic, as different outcomes have been described depending on the host and parasite and study design used [27]. In comparative studies of humans from helminth-endemic regions, worm-infected individuals have different abundances of several bacterial taxa compared to worm-free control subjects, such as increased levels of *Prevotella* spp. as well as increased microbial alpha-diversity (the number of species present within the microbiota; increased diversity has been suggested to correlate with gut health) [28]. However, consistent effects of infection on a core set of bacterial taxa have not yet been demonstrated [29]. Studies utilizing murine models have shown that chronic infection with the whipworm *Trichuris muris* can reduce the alpha-diversity of the GM, and suppress growth and the efficiency of nutrient metabolism [30]. However, infection with *T. muris* or the roundworm *Heligomosomoides polygyrus* can also promote the growth of bacterial genera such as *Lactobacillus* that are normally associated with induction of tolerogenic immune responses, control of inflammation, and improved gut health [31, 32]. Consistent with this, transfer of GM from *H. polygyrus* infected-mice can protect recipient mice against allergic asthma [33]. Moreover, the hookworm *Necator americanus* has been shown to increase GM alpha-diversity during controlled experimental infection in humans [34]. Indeed, a role for the GM has been proposed for the putative beneficial effects of controlled *N. americanus* infection in celiac patients, where hookworm treated patients have reduced symptoms and a higher number of T-regulatory cells and anti-inflammatory cytokines [35]. However, transfer of GM from *H. polygyrus*-infected mice to antibiotic-treated recipients has also been shown to worsen symptoms of infection (colitis) with the bacterium *Citrobacter rodentium*, due to a high level of GM-induced T-regulatory cells leading to reduced bacterial clearance [36]. Thus, depending on context, helminth-mediated changes in GM composition may either have potentially positive consequences resulting from regulation of inflammatory responses, or negative consequences due to impaired nutrient metabolism and metabolic function as well as impairment of appropriate immune responses to concurrent infection with other pathogens.

Whilst studies of the effect of helminths on GM composition and function in livestock are in their infancy, a number of experiments with different helminth species have given some insight into how the GM may respond to infection. During *T. suis* infection, pigs have been shown to have a reduced abundance of a number of bacterial genera in the colon including *Ruminococcus* and *Fibrobacter,* which are involved in carbohydrate metabolism [37]. This may contribute to the reduced growth rates seen in *T. suis* infected-swine [38]. In addition, chronic *T. suis* infection can lead to an increased abundance of *Campylobacter* in the colon, which is speculated to be due to a strong Th2 polarization of immune function impairing effective cell-mediated clearance of opportunistic *Campylobacter* infections [39]. Piglets under field conditions have also been shown to excrete more *Campylobacter* when infected with both *T. suis* and *A. suum* [40]. In experimental infection, pigs infected with *A. suum* have a profound change in the colon microbiome including reductions in *Ruminococcus* and a suppression of metabolic pathways involved in carbohydrate and amino acid metabolism [41], consistent with reports of reduced branched chain fatty acid production in pigs with an acute *A. suum* infection [42]. Notably, these studies also confirm that effects of helminths on the GM are not limited to the predilection site of the infection (the small intestine for *A. suum*), but also can impact the composition of the GM in distal organs such as the colon and caecum.

In herbivorous livestock, somewhat conflicting results have been obtained depending on the samples analysed (faeces vs. intestine), the infection regime, and immune status of the host. A less appreciated factor is also the basal diet of the host, which will likely have a strong influence on the baseline composition of the GM and thus how it responds to infection. In ruminants, a single infection of *H. contortus* in goats was shown to increase total bacterial abundance in the abomasum, thought mainly to derive from a significant raise in abomasal pH [43]. Changes in specific taxa included a significant rise in *Prevotella* spp., a genus also associated with helminth infection in humans and pigs [41], and several predicted metabolic pathways related to lipid metabolism and xenobiotic processing were affected. In sheep infected with both *H. contortus* and *T. circumcincta*, faecal microbiota profiles showed only minor differences over time but did indicate a progressive decrease in alpha-diversity in infected animals [44]. Cortés *et al.* [45] have shown that in young sheep mono-infected with *T. circumcincta*, no change in faecal alpha-diversity was reported but significant increases in abundance of *Prevotella* spp. as well as the putatively pathogenic *Sutterella* spp. genera were detected, indicating a potentially detrimental effect of infection on the metabolic potential and inflammatory state of the gastrointestinal tract. In horses, it has also been reported that faecal GM richness and the abundance of bacterial genera related to carbohydrate metabolism such as *Ruminicoccus* and *Lachnospiraceae* are supressed during cyathostomin infection, accompanied by reduced weight gain in infected animals [46, 47]. In contrast to these studies, cattle that were rendered immune to *O. ostertagi* through serial immunisation by drug-abbreviated infections showed no changes in abomasal microbiota composition following a challenge infection [48]. This may indicate that rapid expulsion of larvae from immune animals is insufficient to drive changes in microbiota, consistent with reports that vaccine-induced Th2-polarized immune changes also do not appear to cause significant alteration of the GM in sheep [45]. However, studies in primary *O. ostertagi* infections with an established adult worm population have not been reported, and further work is needed to delineate the differential effects of acute and chronic infection in parasite-naïve or immune animals.

Collectively, these studies, whilst encompassing significant variation, suggest that helminth infection, particularly in young or susceptible animals, has a substantial effect on the GM. Several core taxa appear to be consistently affected, such as reductions in *Ruminococcus* sp. and increases in *Prevotella* spp.. This may relate to differences in nutrient metabolism reflective of parasite invasion in digestive tissue, as well as alterations in local inflammatory responses due to destruction of mucosal epithelia and modulation of the innate and adaptive immune responses. The nature of these alterations may open up the potential for novel anti-parasitic treatment options based on manipulation of the GM, as well as having implications for host responses to dietary interventions that aim to manipulate GM composition during concurrent helminth infection.

### The influence of dietary components on helminth-gut microbiota interactions

The importance of the GM for human health and homeostasis is now well-established in biomedical science, where microbiota composition and function have shown to impact a range of diseases from diabetes to depression [49–51]. Similarly, in livestock, GM composition has been shown to correlate with a range of health outcomes and performance indicators such as feed conversion efficiency [52]. Thus, targeted manipulation of GM function (e.g. with dietary prebiotics), has been shown to improve livestock health and production [53].

#### Interactions between diet and helminths impact the gut microbiota

The influence of different dietary components on helminth infection has been studied for some time, in part due to the rapid rises in anthelmintic resistant populations and the need for novel control options. The influence of the diet and the digestive environment on helminth burdens was highlighted through studies showing that sheep fed diets containing a high level of plant secondary metabolites such as condensed tannins had lower numbers of parasites, thought to be a result of direct pharmacological binding of the tannin molecules to the cuticle of the worm and subsequent mortality [54]. Subsequently, studies in pigs showed that a high level of fermentable carbohydrates in the diet had an antagonistic effect on worms residing in the colon such as *O. dentatum* [55]*.* In contrast to the pharmaceutical-like effect of tannins, this anti-parasitic effect was thought to derive from the metabolism of the carbohydrates, altering the pH and promoting the production of lactic acid and short chain fatty acid (SCFA)-producing bacteria. This was subsequently confirmed by studies showing that direct infusion of SCFA into the colon of pigs infected with *O. dentatum* has a substantial anti-parasitic effect [56]. This suggested that manipulation of the GM by dietary intervention may be a novel way to control parasites without the use of anthelmintic drugs.

In addition to anthelmintic effects, the notion that dietary components may be used to maintain gut health and mitigate the negative effects of helminth infection is beginning to be explored. In sheep infected with *T. colubriformis* and *H. contortus,* substantial reductions in volatile fatty acid-producing bacteria such as *Ruminococcus* and *Butyrivibrio* were observed in the rumen after infection. However, dietary supplementation with tannin-rich *Acacia* meal reversed many of the differences in ruminal microbiota composition in infected animals, and restored biological pathways within the microbiota predicted to be involved in energy metabolism, despite only a non-significant reduction in worm burdens [57]. This suggests that tannin-rich plants can alleviate some of the negative effects of helminths in small ruminants even in the absence of direct anthelmintic activity. Furthermore, Liu *et al.* [58] have demonstrated that feeding krill oil (rich in polyunsaturated fatty acids) to *T. suis* infected pigs reduced mucosal damage and attenuated the parasite-induced changes in the GM, suggesting that targeted dietary supplements have the potential to restore homeostasis in the face of pathological insult caused by tissue-invasive helminths, and may offer a novel solution to maintain animal productivity in an era of reduced anthelmintic usage.

However, the complexity of the diet-helminth-GM axis is emphasised by recent studies that suggest that some putative health-promoting dietary additives may intensify the parasite-induced changes in the GM, rather than alleviate them. Inulin is a fructooligosaccharide fibre that is easily fermented in the large intestine of monogastric animals and is well-studied in human health and nutrition for its Bifidobacteria-stimulating effects, role in SCFA production, and anti-inflammatory properties [59]. It has also been investigated for its ability to promote gut health and combat diseases such as *Brachyspira* and *Salmonella* infection in pigs and poultry, through a GM-mediated mechanism [60, 61]. However, in pigs given a short-term *T. suis* infection*,* both inulin and infection appeared to have comparable effects on GM composition, resulting in an additive effect of inulin and infection including a decrease in the abundance of putatively harmful bacteria from the Proteobacteria phylum [62]. Dietary inulin also enhanced parasite-induced Th2 immune function and synergistically suppressed pro-inflammatory responses in the colon, suggesting that the natural Th2 polarization was amplified by the changes in GM brought about by the dietary intervention [63]. Notably, dietary inulin, whilst not affecting larval establishment, has been shown to cause accelerated expulsion of adult *T. suis* worms, which may possibly derive from this increased Th2 responsiveness [64]. Similar results have been obtained in pigs fed a prebiotic, grape polyphenol-enriched dietary supplement during the acute phase of *A. suum* infection. In this case, both infection and diet also had broadly similar effects on the GM, including an increase in *Prevotella* and a reduction in *Ruminococcus*, which was accompanied by a significant enhancement of *A. suum*-induced mucosal eosinophilia in pigs fed grape polyphenols [42]. Consistent with the notion that *A. suum* infection may in fact induce some aspects of a ‘healthy’ gut, SCFA levels are elevated in pigs with a long-term *A. suum* infection, and transcriptional pathways associated with inflammation are supressed [33, 65]. These results are seemingly in agreement with some rodent and human models which suggest that helminths can bring about changes in the GM associated with reduced inflammation and increased immunological tolerance. Interestingly, evidence of this has been provided in other experimental systems where anthelmintic treatment of either goats [66] or horses [67] resulted in the subsequent expansion of potentially harmful bacteria from the Proteobacteria phylum within the GM. Consequently, in some situations and species, prebiotic additives that are commonly used in the livestock industry to promote gut health may in fact augment helminth-induced changes in the GM.

The implications of this require further investigation. A combinatorial anti-inflammatory effect of prebiotics and helminths may have some relevance for treatment of inflammatory disorders in human health. Moreover, dietary treatments that may enhance helminth-induced Th2 responses may also aid in the development of protective immunity and worm expulsion. However, in many cases, the profound immunomodulatory properties of helminths may predispose livestock to secondary bacterial or viral infections, and dietary additives that further reinforce this immunoregulatory state may have negative consequences for animal health and productivity. Thus, careful consideration is required to assess the multitude of interactions that may exist between helminths and dietary additives and how these may impact GM and animal health in different contexts.

#### Probiotics and helminth infection

The provision of beneficial bacteria, e.g. *Lactobacillus* (as well as the recently re-named *Lacticaseibacillus*), or *Bifidobacteria* in the form of probiotic dietary supplements is one of the fastest growing sectors of the animal health industry [68]. The question of whether probiotics may be used as a novel tool to control helminths, or conversely, whether concurrent helminth infection may impact on the beneficial effects of probiotics, requires investigation. Some conflicting results have been obtained in pre-clinical rodent models. Administration of *Lacticaseibacillus rhamnosus* (JB-1) to mice was shown to enhance expulsion of *T. muris* in an interleukin-10 dependent manner [69], whereas administration of *Lacticaseibacillus casei* significantly enhanced burdens of the same parasite [70]. In light of the above-mentioned studies showing that helminth infection can favour the growth of *Lactobacillus*, it is perhaps intuitive that administration of probiotic lactobacilli may favour helminth establishment in a reciprocal manner. This was elegantly shown by Reynolds et al. [71] who demonstrated that not only did *H. polygyrus* increase the abundance of endogenous *Lactobacillus taiwainensis* in the GM, but also that administration of exogenous *L. taiwainensis* was sufficient to increase worm burdens when administered together with a primary infection to naïve mice.

Studies on how probiotics may influence the course of helminth infection in livestock are currently limited, although the few studies that have been conducted do not support a role for probiotics as an anthelmintic therapy. Jang et al. [72] showed that administration of *Lacticaseibacillus rhamnosus* LGG (together with a prebiotic cocoa supplement) suppressed the development of Th2 responses during *A. suum* infection, and resulted in delayed expulsion of larvae from the intestine. Similarly, administration of *Bifidobacterium animalis subspecies lactis* (Bb12) resulted in a higher (albeit non-significantly so) number of worms in the intestine of *A. suum*-infected pigs, accompanied by modulation of the parasite-induced mast cell and eosinophil responses in the intestine [73]. This attenuation of Th2 responses is perhaps consistent with the well-known role of probiotics in suppressing symptoms of allergy [74]. Notably, the anti-allergenic properties of probiotics have also been shown to modulate characteristic hallmarks of helminth-induced immune function such as gut hypercontractility in mouse models of trichinosis [75].

Under some conditions, the resolution of Th2 inflammation in the gut of livestock infected with helminths could be considered to have some positive connotations, such as reduced energy expenditure and more nutrient partitioning to growth. Indeed, impaired glucose absorption, a hallmark of the intestinal response to acute *A. suum* infection, was restored in infected pigs fed Bb12 [73]. Similarly, it has been shown that in pigs infected with *O. dentatum,* a mixture of *Enterococcus faecium* and *Bacillus* spp. did not alter worm burdens, but did significantly suppress mucosal inflammatory and Th2 responses in the gut [76]. However, it was also shown that whilst these probiotic strains induced an increase in alpha-diversity and abundance of *Bifidobacterium* spp. in faeces, these beneficial effects were muted during concurrent *O. dentatum* infection, suggesting that helminths also have the capacity to alter the host response to probiotic supplementation. The mechanistic nature of this interaction requires further investigation. Probiotics are known to favour the development of regulatory T-cell responses which may impact on the development of anti-helminth immune mechanisms. How helminths may themselves interact with probiotic bacteria is not clear, but worms such as *A. suum* can secrete anti-microbial substances to influence their surrounding micro-environment [77], and anti-microbial peptide homologues have been identified in other worms such as *Fasciola hepatica* [78]. Intriguingly, worms also harbour their own intestinal microbiota, although how this interacts with the host GM is not yet well understood [79]. Unravelling this complex interrelationship between the host microbiota and ‘macrobiota’ may offer further opportunities to understand how helminths manipulate their host to ensure their survival, and provide future therapeutic avenues.

### Future directions

Numerous studies have now begun to elucidate the complex interrelationships between helminths and the GM in livestock. The challenge now for the parasitology research community is to understand the functional implications of helminth-induced changes in the GM. To this end, increasingly rigorous methodological approaches will need to be applied, including combinations of metagenomic and metabolomic analyses of different gut segments to better understand the localized and systemic effects of helminths and how they impact upon not only the prokaryotic GM but also on the host virome and fungal and protozoan communities. Standardization of the various methodologies used for GM analysis, ranging from DNA extraction to sequencing approach and subsequent bioinformatics analysis, will allow increased comparison of different studies. Ultimately, determining how changes in the abundances of different bacterial taxa relate to changes in host metabolism will be key to future efforts to manipulate the interaction between helminths and the GM, and thereby improve animal health and productivity. Moreover, during natural conditions co-infections between different parasites (e.g. helminths and coccidia or *Giardia*) are often present, and understanding the potential combinatorial effects on the GM is another priority.

Clearly, the impact of helminth infection of the GM is context-dependent, and this context will go some way to defining how changes in diet and other extrinsic factors will modulate the helminth-GM relationship. In some cases, the provision of GM-targeting components such as prebiotics appears to restore homeostasis and attenuate effects of infection, whilst in other cases additive, unidirectional effects of prebiotics and helminth infection on GM composition and immune function have been observed. These findings highlight the intricate interrelationships that exist between the parasite and the host GM and immune system, and that modulation of this system by dietary additives is likely to be a complex and multifaceted process.

## Conclusion

The role of the GM in animal health will continue to be highlighted in the coming years due to increasing pressure on the livestock industry to ensure sustainable and efficient production in an era of lower drug usage and lower carbon footprints. Novel dietary additives to promote animal health are continuously being developed, and several important questions will have to be addressed. Is there any potential for ‘next-generation’ prebiotics or probiotics to be used as a practical tool to control helminth infection in either ruminants or monogastric animals? How important is concurrent helminth infection in influencing the response of animals to nutritional intervention such as probiotics – will these be less effective in helminth-infected animals? Perhaps most importantly, a more detailed understanding of how parasitic helminths interact with the commensal GM within the intestinal environment may shed more light on the complex regulation of the gastrointestinal ecosystem, and allow rational development of tools to progress animal health in the 21^st^ century.

## Data Availability

All data is included in the published article

## Abbreviations

Bb12: *Bifidobacterium animalis subspecies lactis*
GM: Gut microbiota;
ML: Macrocyclic lactone
SCFA: Short chain fatty acid
Th: T-helper.
